# Perceptions and Experiences of a Multimodal Rehabilitation Program for People With Post‐Acute COVID‐19: A Qualitative Study

**DOI:** 10.1111/hex.70283

**Published:** 2025-04-29

**Authors:** Beatriz Carpallo‐Porcar, Sandra Calvo, Sara Pérez‐Palomares, Laura Blázquez‐Pérez, Natalia Brandín‐de la Cruz, Carolina Jiménez‐Sánchez

**Affiliations:** ^1^ Department of Physical Therapy, Faculty of Health Sciences Universidad San Jorge Zaragoza Spain; ^2^ IIS Aragon Zaragoza Spain; ^3^ Department of Physiatry and Nursing, Faculty of Health Sciences University of Zaragoza Zaragoza Spain

**Keywords:** COVID‐19, multimodal program, physiotherapy, qualitative research, telerehabilitation

## Abstract

**Introduction:**

Home‑based rehabilitation has emerged as a practical solution for post‑acute phase COVID‑19 recovery, but patient perspectives on the different modalities remain underexplored.

**Objective:**

To explore participants' perceptions and experiences after a 12‑week multimodal rehabilitation program delivered via asynchronous telerehabilitation versus a booklet after discharge and to identify the preferred format.

**Methods:**

Qualitative descriptive study with two face‑to‐face focus groups of post‐discharge COVID‐19 patients (*n* = 12; age range 41–75 years; 50% female; with fatigue > 4 on the Fatigue Severity Scale) that included participants from each intervention arm of a randomised pilot study. Semi‑structured interviews to determine patients' perceptions and experiences were recorded, transcribed verbatim and coded independently by two researchers using inductive thematic analysis.

**Results:**

Three overarching themes emerged from the analysis: (1) Facilitators for engagement and adherence: Innovative digital tools and personalised guidance foster active participation by providing flexible access and systematic progress monitoring; (2) Barriers to sustained participation: Technological issues, physical limitations and fluctuating motivation serve as critical impediments, underscoring the potential benefits of hybrid intervention models; and (3) Therapeutic alliance as support: A robust, individualised therapeutic relationship enhances patient confidence and self‐management, ultimately contributing to sustained empowerment and recovery.

**Conclusions:**

A multimodal home‐based rehabilitation program with monitoring and personalisation by the physiotherapist is rated positively by post‐acute COVID‐19 patients, with asynchronous telerehabilitation emerging as the preferred method. Future research should investigate long‑term adherence, clinical efficacy and scalability.

**Clinical Trial Registration:**

Clinialtrials.gov #NCT04794036.

**Patient or Public Contribution:**

Post‐acute COVID‐19 patients contributed to the study by actively participating in its development, specifically through describing their experiences as part of a multimodal rehabilitation program. There was no additional participation or contribution from the public to the research.

**Abbreviations**
ATG: asynchronous telerehabilitation group
BRG: booklet‐based rehabilitation group
HEFORA: Health for All Platform
IADL: instrumental activities of daily living
ICU: intensive care unit
RCT: randomised clinical trial

## 1 Introduction

The COVID‐19 pandemic and isolation affected all aspects of healthcare [1] and required a change in attention [2]. Given the strict policy of social distancing initiated by the COVID‐19 pandemic [3], new rehabilitation approaches had to be adapted, and the use of technologies for healthcare was helpful to serve the entire population [4, 5], especially the new COVID‐19 patients who overwhelmed rehabilitation services in 2020 [6].

A particular challenge was the COVID‐19 patients without severe sequelae who were discharged but still required ongoing rehabilitation, without access to face‐to‐face services. A cohort study, which involved 745 individuals, revealed that up to 16% of patients hospitalised during the first wave continued to exhibit rehabilitation needs 5 months post‐discharge. Unfortunately, rehabilitation services were overwhelmed and unable to adequately meet this demand [7].

Multimodal rehabilitation programs have proven effective in improving the quality of life and physical capacity of COVID‐19 patients [8]. These programs usually include a combination of aerobics, strength and respiratory exercises, along with educational components [9, 10, 11]. Other programs employ a multimodal psychosomatic therapy approach that combines individual and group psychotherapy and physiotherapy sessions with a focus on endurance and strength training, as well as interventions such as respiratory therapy or mindfulness and stress management [12].

In the context of COVID‐19, home‐based rehabilitation programs in various formats have already demonstrated their effectiveness in improving health under different conditions [13, 14, 15]. Telerehabilitation provides rehabilitation therapies to remote individuals [16] or people with long‐term rehabilitation needs [17]. For example, the study by Li et al. showed that an unsupervised home‐based 6‐week exercise program delivered via smartphone and monitored remotely with heart rate telemetry led to an improvement in physical capacity and quality of life [18].

There is no formal structure for the delivery of telehealth, and numerous modalities of telerehabilitation can be used [19]. Furthermore, exercise‐based home rehabilitation has been shown to be as effective as real‐time telerehabilitation in treating musculoskeletal and post‐acute COVID‐19 conditions [20, 21, 22]. Thus, telerehabilitation has become a therapeutic window for patients, taking a patient‐centred approach and overcoming geographic, temporal and financial barriers [19]. A systematic review and meta‐analysis found that telerehabilitation could be an effective and safe option for enhancing physical function in post‐COVID‐19 patients. However, further well‐designed studies are needed to continue in‐depth exploration of this topic [23].

Given the well‐established evidence of telerehabilitation, its success and feasibility depend on patients' satisfaction and acceptance as well as overcoming barriers they may encounter [24]. Research suggests that if part of the rehabilitation can be automated, this would allow many patients to be followed up remotely in a more cost‐effective way. These features could make physiotherapists more efficient in following a greater number of patients, as well as allowing the patients to ensure their access to therapy [25], and improve their adherence to treatment [26, 27]. Qualitative research has started to investigate how telerehabilitation is experienced from the perspective of patients [28, 29]. For example, the study by Palacios‐Peña et al. examined the experiences of individuals with post‑COVID‑19 Condition symptoms who participated in supervised telerehabilitation and home‑based respiratory muscle training. The study identified key elements that patients and physiotherapists need to enhance these programs and provided insights into how different users navigate their healthcare context [28, 30, 31].

Nowadays, there is a need for qualitative studies investigating the experience, barriers and facilitators for COVID‐19 patients. Therefore, the aim of this study was to explore the perspectives of patients on a multimodal program performed after hospitalisation, comparing isolated telerehabilitation with the usual format through a booklet. This study was part of a single‐blind, parallel, randomised pilot study with two intervention arms for post‐acute COVID‐19 patients [32]. The research question being addressed was: ‘What are participants' perceptions and experiences after participating in a home‐based multimodal rehabilitation program delivered via asynchronous telerehabilitation versus a booklet and which modality do they consider most appropriate?'

## 2 Methods

### 2.1 Study Design

This study is part of a larger research effort evaluating the efficacy of asynchronous telerehabilitation versus a booklet‐based rehabilitation program for patients recovering from post‐acute COVID‐19 [32]. This qualitative descriptive study was conducted within an interpretative (constructivist) paradigm [33], which assumes that knowledge is co‑constructed between researcher and participant and that meanings emerge through social interaction. Data were collected via focus groups, guided by the COREQ Checklist to ensure the quality of the study [34], and analysed using inductive thematic analysis, allowing for an in‑depth exploration of participants' subjective perceptions and experiences without the imposition of a pre‑existing theoretical framework [35, 36].

### 2.2 Ethics

Ethical approval for the study was granted by the Ethics Committee of Aragón (reference number: PI21/019, current protocol version date 4 April 2021).

### 2.3 Participants

A convenience sample of individuals who had completed the pilot randomised clinical trial (RCT) [8] comparing asynchronous telerehabilitation group (ATG) and booklet‐based rehabilitation group (BGR) was recruited for this qualitative study. Following the conclusion of the 12‐week intervention in early 2022, all eligible RCT participants were invited by the researcher who had developed the pilot study to participate. Participants who expressed interest and met the eligibility criteria were included in the study and signed the informed consent form.

The inclusion criteria were: (1) post‐acute COVID‐19 patients discharged after more than 5 days of hospitalisation; (2) be at least 18 years old; (3) have participated in the aforementioned RCT pilot study and (4) have signed the informed consent. The following exclusion criterion was applied: (1) had not completed the intervention.

### 2.4 Qualitative Study Context

The intervention consisted of a 12‐week multimodal rehabilitation program, which included therapeutic exercises (strength, aerobic and respiratory exercises) divided into 3 progressive intensity levels, and therapeutic education consisting of 3 blocks of health advice. The design of the intervention was based on scientific evidence for these patients [37, 38, 39] and has been clinically proven to be effective [8, 40]. In addition, telerehabilitation systems have been shown to be well appreciated by patients. Patients were required to exercise at home 3 days a week according to the instructions. Every fortnight, both groups received control calls in which each patient was assessed to monitor and personalise the progress of the program [32]. Based on the information gathered in the calls, the physiotherapist adjusted the aerobic exercise time or sets and repetitions of the exercise or changed the exercises to the next level of intensity (between 4 and 7 on the Borg scale).

The ATG delivered the multimodal rehabilitation program via an asynchronous telerehabilitation platform on the Internet, accessible through a mobile app (HEFORA). The program was presented through explanatory videos with a specific description. The platform allowed the physiotherapist to customise the program characteristics. The BRG participants received the same program through a home rehabilitation booklet that included key images and specific descriptions.

### 2.5 Research Team

Given the inherent reflexive and active role that qualitative researchers play in the data collection process, the researcher's positioning was described according to the theoretical framework (Table 1) [34].

**Table 1:** Positioning of the researcher.

Theoretical framework	The only thing I knew about physiotherapy treatment using rehabilitation and telerehabilitation in post‐COVID‐19 patients was through the news, and in more depth during this study. Before the study, I had not had any professional relationship with patients with sequelae of this disease, whether in hospitals, clinics or nursing homes.
Previous experience	I have not been through this disease; my only experience of COVID‐19 is from people close to me and from the media.
Beliefs	I think there is a great lack of knowledge about this disease and all its consequences.
Motivation for research	To generate knowledge about this disease and new perspectives in the field of physiotherapy care.

Also, pre‐conceptions before data collection and throughout the analytical process were documented in a reflexive diary [41]. A regular dialogue was maintained with the research team to discuss the research process and address any epistemological conflicts arising from differing perspectives on nature, source and limits of knowledge and the interpretation of the data.

### 2.6 Data Collection

The data was collected in February and March 2022. At the beginning of the study, two unstructured interviews were conducted on ‘Microsoft Teams’ to determine if the questions were understandable and/or needed to be adapted. The interview guide was developed considering the literature review [42, 43, 44], the research question and the overall purpose of the study. As a result, the interview guide was shortened, and the questions were adapted for the focus groups (Table 2).

**Table 2:** Semi‐structured interview guide.

Subject of research	Question
Post‐COVID‐19 patients' perceptions after the multimodal program	
In relation to the study	− What is your opinion on the physiotherapeutic approach of COVID through platform or booklet? − How did you find the interaction with the physiotherapist? How do you feel about control calls every two weeks? Do you think they are necessary? − How did you feel physically and emotionally during the multimodal program? − Have you been able to do new activities after the rehabilitation program that you could not do before? − Did you encounter any impediments to the multimodal program? − How satisfied are you with the multimodal program, and what aspects would you highlight? − What adjustments do you think could have been made to make the multimodal program more suitable to your needs?
Concerning the post‐study	− Are you still implementing the program? − What are your future goals/perspectives after completing this program?

The second phase consisted of two focus groups with participants from both groups (ATG and BRG) conducted in person, led by a semi‐structured interview guide created with the changes from Phase 1, which served the same purpose and addressed the key areas of the study. The participants had the opportunity to reflect on their experiences with the multimodal program, depending on which rehabilitation modality they were assigned to. To ensure robust methodology, the focus groups included participants from both pilot study arms to gather balanced and comprehensive insights. By comparative exploration of interview data throughout the analysis process, alongside determining that no new aspects, dimensions or nuances of codes were being identified, it was agreed that data saturation was reached [45].

Focus groups were facilitated by S.C., who acted as a facilitator and encouraged an open dialogue through which participants could share and co‐construct the meaning of their rehabilitation experiences. A second researcher (C.J.S.) observed and kept a reflexive journal documenting how the questions and presence of the moderator influenced data generation, enhancing transparency, credibility and rigour throughout the analysis process [46]. A tape recorder was used.

### 2.7 Data Analysis

Data were analysed using inductive thematic analysis [36]. The recorded interviews were transcribed verbatim by L.B.P.; C.J.S. and S.C. reviewed the transcripts for accuracy. Observer annotations were added to the transcripts to complete the information provided and its meaning. The transcripts were inductively coded and analysed independently by two researchers (L.B.P. and S.C.). S.C. started the process by reading and rereading each interview multiple times to allow for immersion and gain familiarity with the depth and breadth of the data. The second phase involved processing the descriptive content to obtain the units of meaning (codes) and attempting to express the data as forms of concepts. In the third phase, the data were deepened to identify the potential themes and sub‐themes. These were then reviewed (Phase 4), defined and named (Phase 5), and finally reported using vivid extract examples [36]. Any disagreements with themes or coding were resolved through discussion with the entire research team. A researcher independently reviewed the sections by comparing the codes and themes to the transcripts. This was a valuable process as it assisted in refining themes [47]. Pseudonymity and confidentiality were maintained by assigning an alphanumeric code [47] to each participant. No software was used for this data analysis.

## 3 Methodological Rigour

To ensure reliability, analysis procedures were applied that considered personal and team interactions as well as research methodological biases, such as transcript review and representation of themes. Validity was maintained through preliminary results checking and the triangulation process. Additionally, fidelity was ensured by collecting data that captured the diversity and complexity of the phenomenon under study. The researcher's perspectives were managed during both data collection and analysis to minimise bias [43]. Finally, a researcher‐informant check was carried out to validate the interpretation of the data collected.

## 4 Results

12 participants who had completed one of the intervention arms of the randomised controlled pilot study were invited to participate in two focus groups. Each group consisted of six individuals: the first included five males and one female, and the second comprised five females and one male. The focus groups lasted 65 and 71 min, respectively. Participants' demographic (age and gender) and assigned intervention arm are detailed in Table 3.

**Table 3:** Characteristics of participants and intervention assignment.

Participant identification (ID)	Gender (F/M)	Age (years)	Hospital situation (acute phase)	Symptoms at discharge	Group assignment
P03	Male	72	19 days in ICU, intubated	Needs assistance in IADLs; moderate fatigue and moderate aerobic capacity limitation	Platform program
P12	Male	51	22 days in ICU, intubated	High limitation of aerobic capacity	Platform program
P16	Female	59	13 days in hospital ward, without oxygen therapy	Needs some help in daily life and severe fatigue	Platform program
P20	Female	41	5 days in hospital ward, without oxygen therapy	Severe fatigue and moderate limitation in capacity aerobic capacity	Platform program
P21	Female	52	5 days in hospital ward, without oxygen therapy	Needs assistance in IADLs; severe fatigue and high aerobic capacity limitation	Platform program
P32	Male	54	22 days in hospital ward, with oxygen therapy	Needs some help in daily life, severe fatigue and aerobic capacity limitation	Platform program
P37	Male	57	20 days in ICU, intubated	Needs some help in daily life, and high aerobic capacity limitation	Platform program
P04	Female	50	5 days in hospital ward, without oxygen therapy	Needs assistance in IADLs	Booklet program
P15	Female	51	20 days in hospital ward, without oxygen therapy	Moderate fatigue and aerobic capacity	Booklet program
P28	Male	75	75 days in ICU, intubated	Needs assistance in IADLs; severe fatigue and high aerobic capacity limitation	Booklet program
P31	Male	73	15 days in hospital ward, with oxygen therapy	Needs some help in daily life, severe fatigue and aerobic capacity limitation	Booklet program
P36	Female	56	5 days in hospital ward, without oxygen therapy	Moderate fatigue and aerobic capacity	Booklet program

Abbreviations: IADL = instrumental activities of daily living, ICU = intensive care unit.

The thematic analysis of the interviews led to three themes and seven sub‐themes that were important from the perspective of the focus group participants (Table 4). It is presented with direct quotations from participants.

**Table 4:** Themes and sub‐themes organisation and illustrative quotes.

Themes	Sub‐themes	Quotes (participant ID, assigned group)
Facilitators for engagement and adherence	Flexibility and convenience	‘If they give me a sheet for my training maybe it wasn't as effective as the mobile phone platform.’ (P37, platform)
	Progress tracking and monitoring calls as a motivator	‘Every two weeks someone is asking you: how are you doing? How are you? … the calls are very good’ (P04, booklet)
Barriers to sustained participation	Technological challenges	‘I had technological problems … but they were quickly resolved.’ (P21, platform)
	Physical limitations, fatigue and motivation fluctuations	‘There are exercises that I can't do, I lie face down on the floor and I can't get up. […] I haven't followed it much.’ (P31, booklet)
Therapeutic alliance as support	Personalisation	‘The physiotherapist adjusted the exercises to my needs … supervision has been very good.’ (P32, platform)
	Post‐program empowerment and recovery expectations	‘I do “Pilates”. It's a way that I've forced myself to do some exercise […]. I have to keep doing some exercise.’ (P15, booklet)

### 4.1 Theme 1. Facilitators for Engagement and Adherence

Despite being randomly allocated to the digital platform or the printed booklet, they adhered to the program because they recognised its critical importance for their recovery. Moreover, an effective therapeutic alliance seems to be a key facilitator for both engagement and adherence to therapy. The digital platform was valued for its interactivity and accountability, while the booklet's clear illustrations and flexibility supported self‐paced use.

‘I think that making it telematic, I think it is because of the profile of the patient, don't you? I think that a higher risk patient perhaps needs to be more directed, more face‐to‐face’.

P20, platform

‘With the booklet it's good, quite good, because there are also pictures, and I see it very well. But well, it looks better on video than on paper, depends on the person. […]’.

P15, booklet

#### 4.1.1 Sub‐theme 1.1. Flexibility and Convenience

Participants described the digital platform as an innovative, intuitive and supportive tool that facilitated their recovery. The on‐demand accessibility of the platform enabled them to complete the prescribed exercises whenever their symptoms allowed, promoting their flexibility and autonomy in their rehabilitation. Users particularly praised the platform's clear, concise instructions and high‑quality video demonstrations for each exercise, which enhanced their understanding, confidence and motivation to adhere to the program.

‘The tool is very good, very well achieved, the exercises are very well explained, and I think it is very positive […], but of course, each person is different’.

P32, platform

‘Although the photos are perfect but, on the video, maybe you can see better how to put it on, how to do it.’

P31, booklet

‘If they give me a sheet for my training maybe it wasn't as effective as the mobile phone platform.’

P37, platform

#### 4.1.2 Sub‐theme 1.2. Progress Tracking and Monitoring Calls as Motivator

Participants identified systematic progress monitoring and regular physiotherapist feedback as key to their engagement and satisfaction. Knowing that their efforts were tracked provided both responsibility and emotional support, enhancing adherence to treatment and overall satisfaction.

‘They encourage you a lot and they are looking out for you.’

P36, booklet

‘I think this program is totally supportive, because, otherwise, at this point I don't think I would still be in rehabilitation’.

P16, platform

Every fortnight, they received follow‑up calls to discuss progress, address issues and adjust exercises. These frequent, empathetic interactions fostered a trusting therapeutic relationship that created accountability, emotional safety and security, thereby strengthening commitment to the exercises. Participants also appreciated the opportunity to share updates and ask questions at any time, and noted the quick, attentive responses they received.

‘Also, I communicated my achievements, I would send her my progress.’

P12, platform

‘I had no problem, I asked the physiotherapist, and she responded quickly, it was fine, no problems, very attentive.’

P04, booklet

### 4.2 Theme 2. Barriers to Sustained Participation

Telerehabilitation enhances participation in multimodal programs by increasing accessibility, scheduling flexibility and patient autonomy, although barriers may reduce adherence and effectiveness. In our study, consistent remote monitoring and personalised feedback mitigated these challenges, underscoring the need for robust support. A hybrid model with initial face‑to‑face assessments followed by ongoing telerehabilitation could optimise engagement and outcomes, provided that the technology and exercise protocols are tailored to each patient's digital skills and physical resilience.

#### 4.2.1 Sub‐theme 2.1. Technological Challenges

Most participants saw telerehabilitation as an innovative tool that offers promising remote care that improves accessibility and self‑management, even with limited digital rehab experience. Occasional technical issues, such as difficulties accessing or loading modules, were resolved immediately and did not affect engagement.

‘I think it's something positive and perhaps innovative, right? Well, it's a support, because I don't think telecare had been done before, right?’

P16, platform

‘I had technological problems, for example, I would load the exercises, but they didn't load’

P21, platform

Several participants acknowledged the benefits of telerehabilitation but recommended a hybrid approach, providing initial face‑to‑face instruction, followed by remote sessions to reduce technological barriers and optimise adherence and effectiveness.

‘If instead of being telematic it was more face‐to‐face or 50%, I think it would be more positive because it would force you to go somewhere to a gym or wherever…’

P32, platform

‘That way I feel obliged to go, because otherwise I don't always have the will.’

P28, booklet

#### 4.2.2 Sub‐theme 2.2. Physical Limitations, Fatigue and Motivation Fluctuations

Participants recognised that therapeutic exercise was essential for recovery and reported reduced fatigue and improved emotional well‑being, even when persistent symptoms and busy schedules made exercise compliance difficult. Many described the time they devoted to exercise as an act of self‑care that changed their attitude.

‘The truth is that both while I'm doing the exercises and afterwards, I feel emotionally pretty good because I'm taking care of myself. It's a time that I'm dedicating to myself.’

P36, booklet

‘They changed my habits, the exercise, they changed the way I look at life.’

P37, platform

However, persistent physical limitations such as the difficulty in performing certain movements served as barriers.

‘There are exercises that I can't do, I lie face down on the floor and I can't get up. […] I haven't followed it much.’

P31, booklet

Early frustration at slow progress was common, but the incremental improvements became a powerful motivator. For a minority, the main benefit of the program was not measurable progress, but a heightened awareness of their remaining limitations.

‘There were days when I felt frustrated because, uh, of course the improvement is long term, and when you see the long‐term effects, it is very motivating’

P20, platform

‘I still can't walk up a hill, I can't walk for 15 min at a time at a normal pace…. So, no, I haven't noticed the effects of the program. But I have become more aware of my limitations.’

P21, platform

### 4.3 Theme 3. Therapeutic Alliance as Support

#### 4.3.1 Sub‐theme 3.1. Personalisation

Participants consistently identified personalisation as the cornerstone of both modalities and described how the exercise prescription was tailored to their individual abilities, symptoms and home environment. In regular follow‑up calls, the physiotherapists adapted the type, intensity and progression of the exercises to the patients' needs, thus promoting confidence, personal responsibility and a sense of safety.

‘To be honest, I felt like it was just for me, that the program it was done to me’

P15, booklet

‘Of course, the physiotherapist adjusted the exercises to my needs, for example, if you can't do the push‐ups, she changed it for another exercise or adjusted the level of intensity. The supervision has been very good for me’

P32, platform

In contrast, some participants would have preferred a face‑to‑face rehabilitation program or at least more comprehensive, detailed supervision, to ensure the right technique.

‘I miss a stronger, more measured physiotherapy recovery. I wanted someone with me at the time to oversee me.’

P03, platform

#### 4.3.2 Sub‐theme 3.2. Post‐Program Empowerment and Recovery Expectations

Upon completing the rehabilitation program, participants described a marked change from passive recipients of care to active managers of their own recovery. Many reported that they had adopted new exercise habits that reflected their growing autonomy and self‑efficacy.

‘It is more about creating a habit for those of us who perhaps didn't have one […] I have continued, not as regularly but, I do try to do it…’

P37, platform

‘I do “Pilates”. It's a way that I've forced myself to do some exercise […]. I have to keep doing some exercise.’

P15, booklet

This behavioural change was accompanied by greater confidence in functional abilities and more optimistic expectations of resuming pre‐illness roles. Several participants noted that the program and enduring therapeutic alliance helped them return to work and daily routines, offering lasting motivation and empowerment.

‘I was stagnating and, well, now I'm going to take the move to start working, I'm not one hundred per cent but that says a lot.’

P20, platform

‘There are many people at home who, if they knew what exercises would benefit them, would do them’

P15, booklet

## 5 Discussion

This is the first study to analyse the post‐acute COVID‐19 patients' perceptions and experiences with a multimodal rehabilitation program comparing two application modalities (telerehabilitation platform and booklet). Patients were satisfied and noted physical and emotional benefits, but desired more initial face‐to‐face sessions. Monitoring, personalisation and explanatory videos were highly appreciated.

The first theme and sub‐themes, although patients reported that adherence to treatment was mainly driven by their own recovery needs, flexibility and the progression‐based program, proved to be the basis for adherence. This can be explained by the fact that telerehabilitation systems (platform or calls) can increase the self‐efficacy of patients, which improves satisfaction and adherence [48, 49, 50], even in people without health literacy, as confirmed by our patients and other studies [51, 52]. Another key for both groups was flexibility: ‘The program can be accessed anywhere on your mobile phone’. Flexible rehabilitation can improve patient comfort and adherence to treatment protocols [53]. For patients with COVID‐19 who have both physical and psychological symptoms, an aspect also highly valued was the flexibility in carrying out their rehabilitation [54, 55].

Participants who used the platform identified the videos as a key facilitator, finding them very helpful and motivating. The videos, in which the patients see themselves reflected, and the objective set on the platform are two tools that increase the internal motivation of our patients. This is very important since one of the difficulties that most concerns patients was the adaptation and preferences of the exercises to their needs [56]. A review indicates that the use of technology, where participants see that the exercises are adapted to them on different levels, improves motivation [57].

The gradual progression of the program through fortnightly monitoring by the physiotherapist also supported the adherence of our participants [58]. Progressive exercises help patients build self‐confidence and maintain their engagement in their rehabilitation programs [59]. Studies have shown that patients who participate in progressive programs are more likely to experience improvements in their physical and mental health [60]. In addition, the integration of technology has been shown to enhance the motivational effect of these exercise programs [59].

Some patients emphasised the personal follow‐up calls as crucial for their adherence to therapy, satisfaction and well‐being. Regular feedback and support play a crucial role in maintaining patient motivation and ensuring the effectiveness of the exercise program [60]. This call system was shown to be equivalent to treatment attendance in the meta‐analysis by O'Brien et al. [61]. This hybrid system was most valued by patients in this study, as well as in other studies that reported a closer relationship with the therapist via telehealth [62]. Establishing and maintaining a close relationship between patients and healthcare professionals is crucial for achieving optimal treatment outcomes and satisfaction among individuals with Long COVID [56, 63]. Human connections were key [64] to creating a therapeutic alliance that encouraged patients' collaboration in recovery [65]. Similarly, another study using telerehabilitation based on exercises through video calls with follow‐up via WhatsApp reported satisfactory experiences in patients who had to stay home due to COVID‐19 [61]. Patients emphasised the importance of physiotherapists listening to them so that the quality of care remains high despite telerehabilitation [66]. These views are consistent with the study by Killingback et al. in which patients emphasised the importance of the telerehabilitation program manager, although in this study, the format was synchronous [67].

In relation to the second theme and sub‐themes, participants felt that despite all these benefits, technological issues, physical limitations and fluctuating motivation serve as critical barriers, highlighting the potential benefits of hybrid intervention models. Between the barriers, the need for face‐to‐face care was demanded. These barriers were only observed at the beginning of the intervention and disappeared as participants became more familiar with the application. Another study investigating patients' preferences for using home‐based telerehabilitation, booklets, videos or an app to guide physiotherapy in older patients found that 59.8% of patients preferred the booklet [42]. In our study, the intervention modality was randomly assigned, and the average age in the focus groups was 61 years. This explains the possible initial reluctance, which disappeared later, suggesting that age is a minor barrier that does not significantly hinder the use of these technologies. In addition, some participants commented on the difficulty of certain exercises, which could be overcome by follow‐up calls.

Regarding the third theme and sub‐themes, the personalisation proved to be one of the keys to intervention in both groups. The presence of messaging and calling systems created a sense of companionship and trust with patients throughout the process. Video calls and rehabilitation phone calls increase the level of perceived social support among patients, which is crucial for their rehabilitation [68]. Phone calls provided a direct personalisation and personal way of interacting, allowing healthcare providers to offer support, clarify instructions and address patients' concerns promptly [69]. This direct communication between therapists and patients enhances the patient–therapist relationship, knowledge and motivation for rehabilitation in orthopaedic patients [70]. The use of telerehabilitation systems to personalise the program increases patient engagement by motivating them to perform their rehabilitation training at home [17]. Another barrier identified in their telerehabilitation program was the lack of follow‐up or monitoring, which led many patients to leave telerehabilitation [31].

Patients also reported physical and emotional improvements after rehabilitation with both systems, which is consistent with the systematic review by da Silva et al. [22]. Many described a transformative physical experience and noted improved health during the multimodal program, which aligns with another systematic review in which patients rated therapeutic exercise highly after COVID‐19 [31]. Improvement in emotional well‐being, self‐care and exercise consistency was also shown, with patients stating that ‘they had changed their habits’. Killingback et al. also suggest that lifestyle habits acquired during rehabilitation should be maintained. Their multimodal intervention, which included therapeutic education on emotional well‐being, self‐care and coping with after‐effects, aimed to increase confidence in home‐based care, which is the first‐line treatment for rehabilitation [71].

Based on a comprehensive analysis of our study's findings, we recommend the implementation of telerehabilitation in conjunction with flexible, personalised programs tailored to the individual needs of patients to enhance existing rehabilitation systems. Regular interactions with healthcare providers via phone and video calls are essential for offering emotional support and strengthening therapeutic alliance. In addition, a hybrid approach that combines telematics with a face‐to‐face system can better address patient preferences while delivering balanced and effective rehabilitation outcomes and experiences.

### 5.1 Limitations

This study has several limitations that affect the transferability and interpretability of its findings. First, the sample was small and recruited by convenience sampling from a pilot RCT at a single centre, limiting generalisability to the broader post‐acute COVID‐19 population. Second, the age range (41–75 years) excluded older adults ( > 75), leading to a potential age bias. Third, voluntary participation after the intervention may have led to self‐selection of individuals with particularly positive or negative experiences. Fourth, the focus group methodology is susceptible to social desirability bias and group dynamics, and no individual member checks or individual interviews were used for triangulation. Fifth, although reflexive diary recordings and team triangulation were used to minimise interpretative bias, the lack of software‐assisted coding and an external audit may have reduced analytic rigour. Finally, the data were collected at a single time point in a specific healthcare context during the COVID‐19 pandemic, so the transferability of these results to other settings or later stages of recovery may be limited by changing treatment pathways and patient needs.

## 6 Conclusion

A multimodal home‐based rehabilitation program with follow‐ups is rated positively by post‐acute COVID‐19 patients, highlighting asynchronous telerehabilitation as a preferable modality than a booklet‐based rehabilitation intervention. Monitoring and personalisation of the program were the two most underlined aspects from all participants' perspectives, being key to the success of both multimodal intervention programs.

## Author Contributions

**Beatriz Carpallo‐Porcar:** conceptualisation, writing – original draft, writing – review and editing. **Sandra Calvo:** conceptualisation, investigation, methodology, formal analysis, writing – original draft, supervision. **Sara Pérez‐Palomares:** writing – review and editing. **Laura Blázquez‐Pérez:** conceptualisation, investigation, methodology, formal analysis. **Natalia Brandín‐de la Cruz:** writing – review and editing. **Carolina Jiménez‐Sánchez:** investigation, validation, writing – original draft, supervision.

## Ethics Statement

Ethical approval was obtained from the Ethics Committee of Aragón (reference number: PI21/019, current protocol version date 4 April 2021).

## Conflicts of Interest

The authors declare no conflicts of interest.

## Data Availability

The data that support the findings of this study are available from the corresponding author upon reasonable request.
